# Exploring attributional and coping strategies in competitive injured athletes: a qualitative approach

**DOI:** 10.3389/fpsyg.2023.1287951

**Published:** 2023-10-27

**Authors:** Federico Leguizamo, Antonio Núñez, Elena Gervilla, Aurelio Olmedilla, Alejandro Garcia-Mas

**Affiliations:** ^1^Department of Psychology, University of the Balearic Islands, Palma, Spain; ^2^Research Group in Data Analysis (GRAD), Department of Psychology, University of the Balearic Islands, Palma, Spain; ^3^Statistical and Psychometric Procedures Applied in Health Sciences (PSICOMEST), Health Research Institute Foundation of the Balearic Islands, Palma, Spain; ^4^Research Group of Sports Sciences (GICAFE), Department of Psychology, University of the Balearic Islands, Palma, Spain; ^5^Department of Personality, Evaluation, and Psychological Treatment, Regional Campus of Excellence Mare Nostrum, University of Murcia, Murcia, Spain

**Keywords:** sport injury, attributions, coping, coping strategies, social support, qualitative research

## Abstract

**Introduction:**

This study explores the attributions and coping strategies of athletes who experienced psychological impact from sport injuries or illness from a qualitative methodology.

**Purpose:**

To understand athletes' unique perspectives on injury and recovery, framed in the Global Model of Sport Injuries, and contribute to the development of effective interventions and support programs for athletes.

**Methods:**

A qualitative research approach was employed, conducting semi-structured interviews with an *ad hoc* sample of 16 athletes, representing diverse backgrounds and competitive levels. Interviews were transcribed and analyzed using NVivo software, identifying themes and codes related to attributions and coping strategies.

**Results:**

Athletes attributed their sport injury mostly to bad luck, routine deviations, and negative mental states, while coping strategies used included cognitive restructuring, emotional calming, seeking social support, mental withdrawal, and behavioral risk. Factors such as training deviations, social support, psychological responses, and injury diagnosis seems to have influenced the coping strategies employed.

**Conclusions:**

Sport injuries and illnesses significantly impact athletes' careers and wellbeing. Support and effective communication from coaching staff and healthcare professionals were identified as crucial for athletes' wellbeing. These findings contribute to understanding the psychological processes and experiences involved in sport injury recovery and highlight key elements for prevention and intervention protocols. Future research should explore communication patterns in sports contexts and assess attributions and coping strategies at different stages of injury recovery.

## Introduction

Sport injuries are an inherent consequence of the practice of physical activity and sport. From a biomedical perspective, a sport injury (SI) is understood as morbid, organic, or functional tissue damage or alteration (DTCM, 1992), produced in the sport domain. Furthermore, for most authors, SI implies impairment of sport practice for at least 1 day (Shultz et al., 2000). From a psychosocial perspective, the SI can have negative repercussions (Forsdyke et al., 2016; Haraldsdottir and Watson, 2021) due to (i) the immediate impact on the health of the injured person, (ii) the interruption of the sport career or (iii) the potential loss of status and social value in athleticism. In addition, SI can seriously affect the psychological wellbeing (Rivas et al., 2012; Liberal et al., 2014) and mental health of athletes when their behavioral repertoire becomes problematic, persisting or worsening over time (Cavanna and Chang, 2016; Gledhill and Forsdyke, 2021).

Due to this psychological component inherent to sports injury, and despite the fact that for some athletes it may represent an opportunity for personal growth (Salim and Wadey, 2019; Rubio et al., 2020), such an event can be interpreted by the individual as a tragedy (Gouttebarge et al., 2019; Chang and Lu, 2020; Leguizamo et al., 2021), as a challenge, or as an escape mechanism in anxious or highly stressful situations (De la Vega, 2003). Although a SI is usually caused by physical factors (lack of physical preparation, overtraining, actions of opponents, etc.), there is a large body of literature that highlights the influence of psychological factors on the risk of injury and in the injury recovery process itself (Andersen and Williams, 1988; Udry and Anderson, 2002; Olmedilla-Zafra and García-Mas, 2009; Gould, 2010; Garcia-Mas et al., 2014; Ivarsson et al., 2017; Goddard et al., 2021).

Brewer's (1994) cognitive model emphasizes the role of cognitive appraisals in shaping emotional responses to sport injury. The model postulates that personal and situational factors can influence cognitive appraisals, which in turn affect behavioral outcomes in the injury rehabilitation process. Situational factors such as injury severity, duration, and impairment of daily activities, as well as life stress, have been found to be inversely related to post-injury emotional adjustment. In contrast, medical prognosis, recovery progress, social support for rehabilitation, physician-rated current injury status, and impairment of sport performance have been positively associated with emotional adjustment. These factors have also been linked to adherence to injury rehabilitation programs, with flexibility in rehabilitation scheduling, responsivity of the rehabilitation environment, social support, and belief in the efficacy of treatment being positively correlated with adherence (Brewer, 1999, 2017). From Brewer's perspective, personal and situational factors might influence the cognitive assessment of a traumatic event (e.g., SI). These factors could greatly determine the emotional response, behavior, and subsequent coping mechanism of the person in that given situation.

Wiese-Bjornstal et al. (1998), based on the stress and injury model by Andersen and Williams (1988), integrated Brewer's model into their own, creating a theoretical framework in which cognitive appraisal mediates between personal and situational factors and emotional and behavioral responses. Later, Olmedilla-Zafra and García-Mas (2009) proposed the Global Model of Sports Injuries (GMSI), a comprehensive model that allows identifying psychological variables as a “galaxy of factors” - a term coined by the authors themselves- that influence SI in three different axes: causal, temporal, and conceptual axis (Olmedilla-Zafra and García-Mas, 2023).

The cognitive assessment made of the SI situation (including beliefs, attributions, and coping) is considered a central axis in these models (Turner, 2016). Rational Emotive Behavioral Therapy (REBT) proposes that negative emotions can be divided into two categories. Healthy negative emotions arise from rational beliefs and are associated with behaviors that help individuals reach their milestones. It is assumed that healthy negative emotions can cause mild physical symptoms and motivate actions that foster goal-achievement (Moesch et al., 2018; Fritsch et al., 2021). On the other hand, unhealthy negative emotions result from irrational beliefs and are associated with behaviors that hinder goal achievement. Unhealthy negative emotions are related to severe physical symptoms and motivate actions that work against these goals. These two types of negative emotions are not just varied in intensity - they differ qualitatively as well and drive different kinds of behaviors (Turner, 2016). This idea is supported by the research showing that so-called negative emotions might be experienced as aversive but are not necessarily harmful. Emotional responses could help direct behavior toward achieving important goals or performing at a competitive level (Van Raalte et al., 2000; Uphill and Jones, 2007). In fact, recent research has challenged the classical negative role of anxiety in competitive sports (Otten, 2009; Núñez-Prats and García-Mas, 2017).

From a behavior analysis perspective, the “irrationality” of hindering beliefs could be explained by referring to the feeling of relief or reduction of discomfort that occurs when performing a particular behavior (e.g., training with pain), and which is maintained by other context-specific reinforcers (e.g., achieving a certain result in a competition while injured or receiving praise from the coach for “giving it all for the team”). Within this framework, REBT's rational and irrational beliefs are seen as learned rules that have been acquired throughout the athlete's history of learning and are prevalent within sports culture, which exert a certain level of control over athletes' behaviors (Martin, 2019). Behavioral patterns that athletes put into practice to regulate their emotional response and manage setbacks, including SI, are referred in the literature as coping strategies (Smith et al., 1990). Coping mechanisms encompass the behavioral strategies that help individuals handle difficult situations (Lazarus and Folkman, 1984; Stone et al., 1988; Folkman, 2008). Within coping, Lazarus and Folkman distinguished between strategies aimed at solving problems (intended to alter or modify the external problem causing discomfort) from those strategies aimed at emotional regulation (regulation of the emotional response to the problem) (Biggs et al., 2017).

People who tend to associate negative events that occur in their lives to internal, stable, and global causes, known as negative attributional style, are more prone to display depressive and detrimental behavior patterns (Peterson and Seligman, 1987; Gillham et al., 2001; Peterson and Bossio, 2001). Whereas, positive or more controllable attributions may act as a protective factor of mental health and performance (Coffee et al., 2009; Cruwys et al., 2015).

The reasoning behind these findings suggests that, in the case of an athlete who suffered from SI, external, stable, and uncontrollable attributions might safeguard self-esteem short-term, but reduce expectations about recovery and increase negative thinking long-term (Roesch and Weiner, 2001). Furthermore, the tendency to associate success and positive events with uncontrollable attributions seems to be linked with helplessness and depressive mood (Abramson et al., 1978; Sanjuán and Magallares, 2007).

Subjective perceptions on the recovery process of SI are related with attribution styles as well. Grove et al. (1990) found that athletes perceived causes of slow recovery as more internal but less stable and controllable than causes of fast recovery. In another study, athletes who perceived they were recovering at a fast rate made more stable and both internally and externally controllable attributions than slow-recovery athletes (Laubach et al., 1996). Positive emotional adjustment is also associated with internal and relatively stable attributions to the cause of SI (Brewer, 1999; Ardern et al., 2012), although other studies have found otherwise (Tedder and Biddle, 1998). Although these discrepancies might be explained by other variables related to SI, such as severity and type of injury, evidence shows that attributions elicit emotions intended to deal with the SI, predisposing behavioral responses with somewhat beneficial consequences in the rehabilitation process. Both problem-focused and emotion-focused coping mechanisms are crucial in the recovery process of sport injury (Brewer, 2017). Consequently, athletes' attributions and coping strategies can significantly alter their behavioral and emotional response, ultimately facilitating or hindering their recovery process. Exploring the affective reactions of athletes toward injury and implementing suitable coping techniques can promote an ideal recuperation and successful reintegration into sports activities (Smith et al., 1990; Clement et al., 2015).

Although evidence-based psychology approaches aim to comprehend how people perceive and make sense of a specific phenomenon, recognizing the unique perspective and specific function of behaviors for each individual, there are fundamental concepts that athletes share and agree upon when they experience a SI (Patton, 1990; Bianco et al., 1999). Qualitative methodologies have been proposed as a reliable alternative to prospectively collect data on the psychological consequences of sports injuries and gain valuable insights into the psychological responses considered in current explanatory models (Brewer, 2007; Mosewich et al., 2013; Smith and McGannon, 2017). The main objective is to gain insight into how athletes who suffered a severe impact injury or illness attributed meaning to their experience and coped with the injury and recovery process.

## Materials and methods

For this study, a qualitative interview methodology based on a phenomenological perspective was deemed as the most suitable approach, following the Standards for Reporting Qualitative Research (SRQR) checklist (O'Brien et al., 2014). Qualitative studies are recommended to include a minimum sample size of at least 12 participants to reach data saturation (Guest et al., 2006; Clarke and Braun, 2013; Fugard and Potts, 2014). Therefore, a sample of 16 athletes (eight women) was deemed sufficient for the qualitative analysis and scale of this study. Considering the evidence-backed resemblance between the sport injury and illness (Bianco et al., 1999), it was determined that athletes who had been through either of the two incapacitant conditions would be eligible for the study.

Given the exploratory characteristic of this study, the sample was selected by opportunity among the co-authors, from different origins and diverse levels of competitive activity. The term “psychological impact” referred to any sport injury or sport-related illness that interrupted sport participation for at least 1 month. Serious events were likely to be significant for the athletes and, therefore, appropriate to explore the psychological processes associated with sport injury and illness.

Table 1 presents a comprehensive overview of the participants involved in this study. Two elite athletes who achieved professional status allowed the examination of the psychological implications of injuries on highly skilled and accomplished athletes. One participant faced the additional challenge of coping with an incapacitating illness, enabling exploration of how athletes manage the psychological impact of such conditions. Three participants experienced a period of uncertainty during their injury recovery process, where the diagnosis of their injuries remained unclear. Only one athlete had no prior experience with sport injuries. This participant's story offered a unique viewpoint, allowing exploration of the psychological adjustment process athletes undergo when confronted with an injury for the first time. Despite the sample being chosen by opportunity, all participants met the inclusion criteria of having experienced a high psychological impact due to their respective injury or illness.

**Table 1:** Demographics on study participants.

**Athlete**	**Sport discipline**	**Clear diagnosis**	**Injury type**	**Experience with sport injury**	**Gender**	**Severity**	**Competition level**	**Current injury state**
1	Trail running	No	N/A	Yes	Female	High impact injury	Competitive athlete	Injured but active
2	Basketball	Yes	Joint/tendon	Yes	Female	High impact injury	Competitive athlete	In readjustment process
3	Tennis	Yes	Joint/tendon	Yes	Female	High impact injury	Competitive athlete	Injured but active
4	Rugby	Yes	Joint/tendon	Yes	Male	High impact injury	Competitive athlete	Fully recovered
5	Football	Yes	Bone	Yes	Female	High impact injury	Elite athlete	In readjustment process
6	Golf	Yes	Joint/tendon	Yes	Male	High impact injury	Competitive athlete	In readjustment process
7	Tennis	Yes	Muscle	Yes	Male	High impact injury	Competitive athlete	Injured but active
8	Athletics	Yes	Muscle	Yes	Male	High impact injury	Competitive athlete	Fully recovered
9	Trail running	No	Joint/tendon	Yes	Female	High impact injury	Competitive athlete	Fully recovered
10	Athletics	No	Joint/tendon	Yes	Male	High impact injury	Competitive athlete	In readjustment process
11	Rugby	Yes	Joint/tendon	Yes	Female	High impact injury	Competitive athlete	In readjustment process
12	Rugby	Yes	Joint/tendon	Yes	Male	High impact injury	Competitive athlete	In readjustment process
13	Rugby	Yes	Joint/tendon	Yes	Female	High impact injury	Competitive athlete	In readjustment process
14	Golf	No	Bone	Yes	Female	High impact injury	Competitive athlete	Fully recovered
15	Cycling	Yes	Bone	No	Male	High impact injury	Competitive athlete	In readjustment process
16	Football	Yes	N/A	Yes	Male	Incapacitant illness	Elite athlete	In readjustment process

A panel of experts trained to conduct the interviews with injured athletes developed a semi-structured interview based on theoretical frameworks related to attributional process and coping strategies (Kim et al., 2003) according to the Delphi methodology. All athletes were asked to state their gender, sport discipline and competition level, history of injury, diagnosis of the high impact injury or illness experiences discussed, the severity of their injury or illness, and current state of their injury. Following the demographics, athletes were asked open-ended questions to allow them to express their experiences with minimal direction from the interviewer. The interviews were carried out in person and through the Zoom telematics platform (Zoom Video Communications Inc, 2022) with an approximate duration of 30 min. In both cases, the interviews were recorded to be transcribed later with Google AI (Google, 2022). The athletes' informed consent was requested in writing to record the interviews.

### Data analysis

Qualitative content analysis was carried out using the NVivo software (QSR International Pty Ltd, 2022) to facilitate subsequent data analysis.

Themes or codes were identified first in relation to attributions and coping strategies through a thorough review of relevant literature and consultation with experts in the field of sport injuries. These themes were then grouped together and analyzed for emerging patterns, consistency, variability, and the function and effects of specific discourses. The interpretation of these themes was conducted through a process of repeated reading and analysis of transcribed audio recordings. Any piece of text that demonstrated one of these themes was categorized under the relevant code(s). During this process, attention was also paid to the identification of new codes.

## Results

A variety of relevant SI attributions and coping strategies influencing the recovery process were identified in the interviews with the athletes. Table 2 presents the list of identified attributions made by the athletes with its definition and select quotes extracted. Avoid injury refers to taking appropriate measures to prevent injury. Warm-up or routine deviation suggests that a lack of proper warm-up or deviation from the athlete's training routine might have increased the risk of injury. Bad luck suggests that the injury may have occurred due to chance or unfortunate circumstances. Finally, an athlete that attributed their SI to being in a negative mental state considers their psychological response during training or competition, such as anxiety or external concerns, as a possible contributor to injury.

**Table 2:** Attributions on cause of sport injury.

**Attributions**	**Select quotes**
Avoid injury (Yes)	*Yes, I believe that as athletes, we are not taught much about how much our emotions influence our injuries. [...] These are extreme training situations, and stress finds a way to manifest. In my case, I feel that my first plantar fascia rupture happened because I wasn't emotionally well, and that triggered constant pain for 8 months. The pain never stopped, but I kept playing, enduring it all. Yes, if I had more resources [...] like being able to afford a physiotherapist every week, having more time for gym workouts, and also work. Time is limited. Certainly, yes. Also, at that time, I had involved myself in another sport [...]. And, of course, I started losing muscle mass in quotes, and I wasn't strengthening or doing injury prevention exercises. Yes, I could have controlled it. I was very excited. I had been training six times a week for 3 months, doing double sessions some days, and pushing myself to the maximum volume required to perform well on the competition day. I believe I should have regulated it a bit more in the weeks leading up to the injury because the body doesn't function properly under constant tension. But well, the desire always prevails*.
Avoid injury (No)	*If I could do something, I did it because I know I usually have problems in my hips and back, especially in the lower area. So, I always do rehabilitation exercises before and after, and stretching. Whatever I could do, I did. Well, I mean, I think, well, I think one of the nice things about sports is that sometimes there are factors that we... that we can't control. And I think one of them is [this type of] injuries. Honestly, no, because you can expect or at least, I always expect the best but prepare for the worst, in a way, that anything can happen. I was very clear about that, that ligaments, ankles, anything could happen, but in life…*
Warm-up/routine deviation (Yes)	*Yes, I believe that has been the main problem. I should have focused more on increasing my muscle mass first and then on pre- and post-training preparations, whether with ice or exercises, all these things. It could be, yes. Most likely, on the day of the injury, I didn't activate myself well enough for the competition*.
Warm-up/routine deviation (No)	*No, because I was very aware, I felt that the upper part was overloaded and I was very aware of the need to warm up properly, progressively. Well, since I'm part of a team, warm-ups and post-training sessions are very controlled. So, we have a warm-up routine that we do every day exactly the same, which lasts 15–20 min before training. I don't think it was that. I have a warm-up routine that is always very similar, besides the team's overall warm-up, which is almost always the same. I've been in several teams, mostly two. The warm-up is always the same. Since this injury happened, I focus much more on warming up my shoulders. Before, it may have been something a bit more secondary, and I didn't give it as much importance. Now I focus on it much more. No, not that, the warm-up part, I mean, on the day when the pain started and such, no, because I always warmed up with my routines, they're always the same, and I always do a lot of warm-ups. No. It was just a normal day. Same routine, same warm-up protocols, everything was planned. Nothing new. In fact, I was already finishing the training session*.
Bad luck (Yes)	*I don't know if it's my case, but it's true that I think that maybe when you let yourself go a little, when you play at a high level, high intensity, you can feel it. I don't think it was my case because I believe that at that moment I was in pretty good shape. I think it was simply bad luck. Yes, absolutely, 100 percent. I think it was an unfortunate incident. Also, I don't think it was my fault [...] for trying to cover something that wasn't really my responsibility, trying to reach something I couldn't reach, it happened to me. It depends on how you look at it. I had bad luck in missing many matches, in missing important moments on the court, in not being able to be there... Sometimes, yes, I did think about it. I believe that luck is sometimes a factor. Yes, yes (laughs). Someone who relies so much on injuries obviously has to reconsider what they are doing wrong. But obviously, once you have reached a quota of injuries, you tend to think that even if you do things right, there must be some bad luck involved, otherwise, you can't explain it. It's a bit frustrating in that sense, but well, as I said, there are things that I could foresee that were not bad luck and other things [...] that were*.
Bad luck (No)	*Well, bad luck (laughs). I think that... In a way, I know I shouldn't complain because in the end I've been lucky, but I think it's a bit unfair, really unfair because I've met many colleagues in my career who maybe, I don't know, they didn't... I'm not asking for anything to happen to anyone, but maybe they didn't take care of themselves at the level I did, they didn't prepare themselves at the level I did, and well… something that doesn't usually happens ended up happening to me. You know?*
Negative mental state (Yes)	*When you have external concerns, external problems, your body releases stress in some way. And especially as athletes, we're constantly demanding 100%. It's not like you can take a break, stay resting at home because you feel bad or have a family problem, a love problem, or whatever it may be. No, you have to keep going, you have to keep pushing yourself. Your body continues to be under 100% demand, and there's no way out of that. So, it's like I feel that my injury was completely emotional. Yes, yes. It's true that sometimes, if your mind isn't 100% focused on the competition, which is where I got injured, then you can be a bit more tense, not execute the correct technique, and that leads to getting injured. Yes, I was going through a tough time. I had finished my studies and couldn't find a job in my field. I was a bit frustrated. It could be. I believe, as I mentioned before, that when your mind isn't fully present, your body notices it. When you're not focused on what you're doing, obviously you don't perform the technical movements as you should, you don't pace yourself properly, you don't maintain the right running technique, and you're also not fully focused on what you're doing*.
Negative mental state (No)	*Well, when I'm training I usually don't bring external things with me because when I play, whether it's in training or in matches, it's like I enjoy it so much that I disconnect from everything happening outside. I don't usually get nervous. External things don't affect me that much. No, not this time, but I think it does have an influence. In fact, the first month after returning, even the first 2 or 3 months were very tough because it made me feel like a much worse player. I wasn't as engaged in physical contact. I don't think so because at that moment, I was very motivated. I really wanted to play. No, I don't think it was due to the mental aspect because I was feeling great at that time when the injury started, and I was performing well in competitions and everything. No, not at all. If there was one thing I was clear about, it was that it was my game. As I said, throughout the year, I couldn't be there, and that was going to be my game, and I was feeling good. No, no, no, no. Because when I say, when I train, it's like I escape, it's an outlet for me. And if it's... if it's a 3-hour training session, then I'm focused on those 3 h. I put aside things like studies, work, etc. No, no, no, in the end, I did feel some tension, but I think tension is good as long as it's controlled. So, I was achieving a little bit of what I had dreamed of all my life, you know? So, there was some tension, a bit of nervousness, but I don't think it had anything to do with it because I've always been able to control that very well*.

Table 3 refers to the different coping strategies athletes employed when faced with adversity. Cognitive restructuring involves reorganizing one's behavior and thought processes to effectively address adverse situations and achieve specific goals. Emotional calming strategies focus on managing and regulating unpleasant emotions that arise during difficult circumstances. Seeking social support is a coping mechanism where individuals seek emotional comfort or practical assistance from others. Mental withdrawal refers to behavioral disengagement or detachment from the situation at hand. Behavioral risk may arise when individuals pursue specific goals in adverse circumstances, which carries the potential to hinder the recovery process.

**Table 3:** Coping strategies used by injured athletes.

**Coping strategy**	**Select quotes**
Cognitive restructuring	*I decided to participate in uphill races, which I can manage, although I've always been bad at them. I told myself, “I'm going to run them. Period.” I did well, and that's when I started to see a glimmer of hope and good news. This will make you a better professional. You'll be able to develop projects focused on rehabilitating other athletes. It's a bit of a mantra I use when I don't feel like doing anything. It's important to understand that this type of pain and situation is not permanent. At first it was harder, but then you start to think and realize that it's all a learning experience and that in the end it makes you stronger. To be honest with you, I think I was doing everything I should have been doing to be okay and yet it happened to me. I take it more as a learning experience, as something that will help me in my future and present as an athlete and as a non-athlete. I have taken the best out of it, I have become a better person, a better athlete, my habits are much better than they were before when I wasn't injured. So I said no, I'm not going to do it that way. As I study what I study, I'm going to prepare myself in case I can compete in July. That helped me a lot because even though I didn't see improvements in my injury, which there have been at times, I have been improving my marks. There are people who live like that without an operation, with torn ligaments and still live a normal life. That could be me, you know? I decided that no, if I had come out of two surgeries, a muscle tear was not going to stop me, and I was going to finish the season and recover. It was a tough week, but I ended up getting through it. Knowing that the situation and illness were difficult but feeling good physically, I said, “Well, if I'm okay in my daily life, why should I worry about what I have? Take care of yourself and that's it.” I was working in a restaurant with some friends and it went very well for me, plus I considered it as a kind of rehabilitation because I had to carry a tray on my shoulder, on the bad shoulder, and of course, I took it as positively as I could. This injury helped me a lot because I had many personal things to do that I wasn't doing. I had to work on them because they took away my sport. And then I started working on myself too. And it was also emotionally hard, but now I'm super happy because I can manage everything that used to affect me more, much better. I always thought I was mentally very strong, but then I needed to seek help from a psychologist. “I'm no longer able to get out of this emotional and physical hole on my own. I need help and I think the mental part is the only thing I can improve on.” That made the difference*.
Emotional calming	*Being in nature improved my mood. Going [...] close to the mountains would calm me down. Everything related to nature had that effect on me. I would analyze the pain, what kind of pain it was and how I felt it. It was like trying to connect with this type of pain, to say, “okay, you're here, that's it. You can't run away from it; you can't escape it. Accept it, you know?” Maybe I was able to accept it for 15 min a day, but that was it. Watching the sunset gives me inner peace, the ability to be with the people who appreciate you. Going for a swim in the open sea also helps me, in a way, it's calming*.
Seeking social support	*Talking to [injured] people that said they haven't felt so much pain. When I was feeling a bit better, I called my sister, or my grandmother would come. Thanks to my roommates I was able to solve a lot of things, because at first, I couldn't even cook. Going with the group [teammates] helped me the most. You have to try to go with your team, even if you do less things because that pumps you up. I called close contacts to ask what you should do when you have shoulder surgery and then they gave m solutions. It helped me to calm down a bit and know what to expect. Something very gratifying was every time the door of the room opened; it was like a breath of fresh air. To see that door and see a friend, a family member... that is priceless*.
Mental withdrawal	*This emptiness changed my relationships with people without realizing it, and I lived a lot inside my mind. It was like I was inside a bubble. Inside my head. I thought about everything. I got frustrated inside my head. I was feeling so bad that I wanted to be alone. Breathing and trying to escape the pain, thinking, imagining things, not physically, not practicing imagination, but escaping, like going somewhere else, other than here. Music helped me a lot to distract myself. When you are in a lot of pain you distract yourself with whatever. Meditation helped me a lot to feel better. To take that habit of stopping, of putting my mind blank, of not thinking about why or why not, what I do, what I don't do. The world of breathing opened up to me, focusing on being calm, not thinking about the injury but about beautiful things, peace, whatever. I took walks and paid attention to the movements and routines of the doctors. I focused on people's gestures, if they are friendly, if they say thank you. And that helped me to be a little more dispersed in the hospital*.
Behavioral risk	*My first injury triggered a pain that lasted for 8 months, without stopping hurting, without stopping playing, trying to endure everything. I had a lot of pain for 2 min, but then it went away. The coaches told me to come out, but I refused. I got up to continue, but I fell again because my knee couldn't hold me up. But I wanted to keep going. I kept playing, obviously in pain, but I'm not usually one of those people who doesn't play when it hurts. So I was taking medication and all that, but I kept playing for2 more months after the injury. When I warmed up and put on some balm, it didn't hurt, so I could compete. But when I cooled down again, the pain came back. That competition went well for me, and I had 4 weeks left until the national championship, so I pushed myself. That's when I got injured. My recovery was going well, and I went back to the training field to run and see my teammates. I felt like I wanted to be there with them, and since I couldn't, I started to run more than I should have. I pushed myself too hard and damaged my ligaments again. That was also very tough. I didn't know what I had, and no one believed me. I had to play tournaments, sometimes with incredible pain, because retiring has never been for me. The situation was getting worse, no one believed me, and I kept training the same way*.

### Sport injury attributions, psychological responses, and coping strategies

Half of the athletes interviewed said their sport injury or illness could have been prevented and most of them attributed their SI to bad luck. However, some athletes attributed their SI to routine training deviation, being in a negative mental state, and warm-up deviation. Regarding coping strategies (CS), most athletes referred to seeking social support, mental withdrawal, and cognitive restructuring in their injury and recovery process. Frustration and sadness were the most frequently reported emotional responses (ER). Additionally, athletes expressed confusion, encouragement, anxiety, discouragement, and disappointment. Concerns about the future, acceptance of their circumstances, and concerns about their sports career were the most reported cognitive responses (CR). Furthermore, athletes reported resignation and thoughts about quitting their sport career as other relevant cognitive responses (see Table 4).

**Table 4:** Proportions of sport injury attributions, psychological responses, and coping strategies.

**Categories**	**Proportions (%)**
Sport injury attributions	
Bad luck	68.75% (11/16)
Avoid injury	50% (8/16)
Routine training deviation	37.5% (6/16)
Negative mental state	37.5% (6/16)
Warm-up deviation	18.75% (3/16)
Psychological responses	
Frustration	93.75% (15/16)
Sadness	68.75% (11/16)
Confusion	43.75% (7/16)
Encouragement	43.75% (7/16)
Anxiety	37.5% (6/16)
Discouragement	31.25% (5/16)
Disappointment	18.75% (3/16)
Cognitive Responses	
Concerns about the future	75% (12/16)
Acceptance	62.5% (10/16)
Concerns about sport career	50% (8/16)
Resignation	37.5% (6/16)
Thoughts about quitting	31.25% (5/16)
Coping strategies	
Seeking social support	81.25% (13/16)
Mental withdrawal	75% (12/16)
Cognitive restructuring	62.5% (10/16)
Emotional calming	50% (8/16)
Behavioral risk	50% (8/16)

Factors such as deviation in training or warmup routines, sport-related or unrelated stressors, or bad luck were associated with the use of different coping strategies for adapting to the recovery process. Athletes who believed their SI was avoidable and caused by deviation from training or warm-up routines tended to exhibit behavioral risk (CS), confusion (ER), and resignation (CR). In addition, athletes who attributed their SI to a negative mental state tended to express more anxiety (ER) and thoughts about quitting their sport discipline (CR). On the other hand, athletes who did not attribute their SI to routine or warm-up deviation and considered it as unavoidable and due to bad luck tended to use cognitive restructuring and emotional calming (CS) to cope with their injury. In addition, they expressed frustration (ER) with their situation, eventually reaching acceptance (CR) of their injury or illness.

As one athlete expressed in relation to the use of cognitive restructuring as a coping strategy:

Now I set mini goals for myself, right? Like going from two crutches to just one crutch, being able to stand up as many times as I want without pain, being able to go upstairs... I don't feel like meeting with people that much, but being able to go out to grab a coffee, you know? That's what motivates me the most. And that's why I write things down, because if I don't, when I look back, it seems like I haven't achieved anything, that I'm still the same. But if I consider the progress that I am making... going upstairs with two crutches, then with one crutch... it's like, “Okay, you are making progress.” And this is essential for me because if my mood goes down, then I try to think objectively to improve my self-confidence, you know? Because otherwise, it's going to be too much for me.

Cognitive restructuring, emotional calming, and seeking social support were the most common coping mechanisms used in the recovery process. In addition, cognitive restructuring strategies and emotional calming are frequently intertwined and interrelated in the athletes' narrative. During the interviews, women highlighted the role of emotional calming strategies, whereas men emphasized the significance of seeking social support.

Another athlete reflected on their experience and the use of coping strategies to deal with unpleasant psychological responses:

Being honest with you, I believe I was doing everything I should have been doing to be okay, and yet it happened to me. I take it more as a learning experience, something that makes me stronger and will help me in my future and present as an athlete and as a person... I prefer to think this way rather than to think, “Oh, why is this happening to me when I do everything right?

### Perception of social support and behavioral risk

Social factors (e.g., lack of perceived support, feeling misunderstood by their peers, pressure to speed up their recovery process) and uncertainty (e.g., unclear diagnosis) were linked to engaging in behavioral risk. In addition, behavioral risk seems associated with the attribution of SI to routine or warm-up deviation. Athletes who reported receiving social support, coaching staff support, and did not attribute their SI to being in a negative mental state reported less or no engagement in behavioral risk.

As one athlete shared the setbacks encountered, they explained how they engaged in behavioral risk:

During rehabilitation, when I was doing very well, I returned to training just to run around and watch the girls play. Of course, in the end, I wanted to be there, and being unable to... I started running more than I should have. I pushed myself too hard. And then my knee said, “That's it” And then mentally I said, “Well, we've reached the limit. I'm not going to run again. I'll never recover.” And that was also very difficult.

The most difficult period for injured athletes was after being diagnosed (5/16), when the injury took place (3/16), the rehabilitation (3/16), waiting for the surgery (2/16), post-surgery at home (2/16), and post-surgery at the hospital (1/16). Besides, unclear SI diagnosis and lack of physiotherapy support was associated with experiencing a disconnection from their sport discipline (i.e., not wanting to hear anything about their sport) and referring to rehabilitation as the most difficult recovery period. Athletes who expressed these ideas felt pressure to speed up their recovery process, engaging in behavioral risk. Although the majority of athletes received some form of social support, a small subset reported feeling misunderstood by their peers regarding the challenges they faced.

### Themes derived from the interview experience

During the course of the interviews, three themes emerged that were not initially covered but were deemed important by the athletes themselves to understand their injury and recovery process experience (see Table 5).

**Table 5:** Themes derived from the interview.

**Categories**	**Proportions (%)**
Relief from sport obligations	25% (4/16)
Seeking psychological help	18.75% (3/16)
Concerns about medication	18.75% (3/16)

The first theme was related to concerns about the use of medication to cope with pain after surgery. As one of the athletes interviewed stated:

As an anecdote, I was forced to take painkillers. I hate medicine. It's just not my thing. Nowadays, we are so used to taking pills for any kind of pain. I wanted to be an active part of my recovery process, but after my operation, I was forced to take pain medication. There's a phrase I like a lot [common in the sports field]: “We're pain junkies.” I was in pain, but I endured it. The only thing that bothered me was the difficulty in sleeping. That's when the doctor came and said, “There are no heroes here.” And he gave me a dose of painkillers.

The second theme involves interest in seeking psychological help. Some athletes reflected on the importance of counting with professional psychological support to learn how to cope with their injury.

I tried… I thought many times about seeking out a specialist to talk about it, but it never happened because ultimately, despite all the feelings I expressed about sports or the frustration I had experienced for years, I felt like even if I had a specialist from the beginning who would have taken things step by step, they might not have understood me. Whether it was because of my personality or how I am or am not, I decided to make the decision on my own.

While no athlete perceived being injured as a positive condition, some reported experiencing relief from the demands of high-performance training and competition during the recovery process.

I always say that you must look for the positive side. Well, now that I have more time, I have time to go out with friends to grab a drink, an ice cream, and watch the sunset, which I'm a fan of, and that gives me inner peace, the ability to be with people who appreciate you. The fact of going out for a walk, even if it's just to find that calmness watching a sunset, for me that is... it's spectacular!

## Discussion

Sport injuries and illnesses are an inherent part of sport practice that often come unexpectedly and might impact the career of athletes. Injuries are a common and often unavoidable occurrence in sports regardless of an athlete's characteristics. Each athlete's injury experience is unique and requires individualized psychological intervention. Therefore, not all injuries have the same effect on athletes; in general, serious or very serious injuries that require extended rehabilitation periods typically result in more negative experiences for the athlete. These outcomes can include a halt to their sports career, emotional and familial disruptions, economic loss, irreversible physical damage, and even quitting sports altogether. A serious injury can have significant and long-lasting effects on an athlete's economic, professional, physical, and psychological wellbeing, and its consequences can have repercussions on the rest of their life (Wojtys and Brower, 2010; Wiese-Bjornstal, 2014).

In the present study, stories shared by athletes from different sport disciplines and competitive levels support that (i) cognitive restructuring, emotional calming, and seeking social support were the most common coping mechanisms used in the recovery process; (ii) athletes' attributions regarding their injuries can impact the recovery process, as well as partially determine the coping strategies implemented during this period; and (iii) social support, among other external factors, could influence the engagement in behavioral risk. The impact of the SI in the life of athletes, beyond the attributions made, is reflected in the experience of the recovery process, as a difficult period remarked with feelings of frustration, anger, sadness, uncertainty, and devastating thoughts. Among the derived themes, athletes expressed concerns about taking medication, interest in requesting psychological support, and feelings of relief from their sport demands during their recovery process. These findings have important implications for sports coaches, trainers, federations, and health practitioners.

Understanding how athletes attribute causes to their injuries, their experiences during the recovery process, and their coping strategies can help them and their support networks to better manage the SI recovery, as well as potentially prevent future injuries (Nicholls and Polman, 2007; Crocker et al., 2015; Olmedilla-Zafra et al., 2017). Regarding the attributions made by the interviewed athletes, most athletes attributed SI to external and unexpected factors categorized as bad luck, while others attributed SI to internal factors such as a previous altered emotional state and a deviation from their sports routine. Among the latter, and in line with previous research (Weiner, 1972; Roberts and Pascuzzi, 1979), some believe that they could have prevented the SI since they had some degree of control over the circumstances of the incident. The process of dealing with a sports injury can be broken down into stages, as described by Olmedilla-Zafra and García-Mas (2009, 2023), where the recovery period usually involves acceptance and adaptation to the new situation. Interviewed athletes demonstrated various coping strategies to manage their sports injuries (SI). These strategies included engaging in reappraisal of the injury and its contextual repercussions, seeking social support, participating in leisure activities, and adopting alternative roles to maintain their connection to the sport discipline during the recovery process. It is worth noting that the duration and resolution of the SI greatly influenced the psychological impact experienced by the athletes and the coping strategies they employed.

While these semi-structured interviews initially aimed to explore the attributions and coping strategies of athletes with SI diagnosis, it is important to consider the derived (aka emerging) themes highlighted by the athletes themselves. Some expressed concerns about the consequences of their injury on their mental health, reflected in doubts regarding whether they should talk to a psychologist or if the medication either administered or prescribed to them would have detrimental consequences. Even though most athletes received support from healthcare professionals, some of them regretted the lack of communication and suffering due to unclear diagnosis. One athlete admitted to receiving abusive comments from medical personnel regarding their recovery progress, and another decided to quit their sport career largely due to medical malpractice and their failure to provide support. Lastly, athletes reported experiencing a certain degree of relief from the pressure of their training and competition duties, also observed during the competition and training pause due to the COVID-19 pandemic (Leguizamo et al., 2021).

The combination of an unclear diagnosis and a perceived lack of support from coaching staff or medical professionals appears to significantly contribute to experiencing the rehabilitation process as the most difficult stage of SI recovery. Athletes who encountered these circumstances reported upsetting experiences, unpleasant feelings, and engaging in behavioral risk.

One athlete recalled a personal situation that reflects the latter:

I didn't know what I had, and no one believed me. I had to play tournaments, sometimes with incredible pain, because retiring has never been for me. The situation was getting worse, no one believed me, and I kept training the same way.

Furthermore, athletes' perceptions of the cause of their injury may influence their emotional, cognitive, and behavioral responses. Those who view their injury as avoidable may feel frustrated with themselves or their adapted training routine, leading to behavioral risk as they try to make up for lost time. According to previous research, this counterproductive way of coping with injury might explain the positive correlation between use of behavioral risk and number of injuries (Rubio et al., 2014). Athletes who believe a negative mental state may have led to injury could have felt overwhelmed and anxious, leading to thoughts of quitting their sport. Social support from coaches and teammates may reduce engagement in behavioral risk, decreasing the chances of athletes interfering with their recovery plan due to psychological factors (Bianco, 2001).

Attributions related to the perception of control over the outcomes of athletes' behavior has been traditionally categorized by the scientific literature as internal or external locus of control (Rotter, 1966; Lazarus and Folkman, 1984; Wang et al., 2022). Internal attributions reflect results as the consequence of one's own actions, while individuals with external attributions perceive results as being influenced by external factors, which can be applied to use of coping mechanisms (Lazarus and Folkman, 1987; Folkman, 2008). However, it is argued that these broad measures need to be more specific to situations or contexts, since the environment in which emotional responses are elicited might moderate these attributions (Uphill and Jones, 2007). An individual's general locus of control may not always be highly correlated with their behavior in specific situations, suggesting the importance of considering other situational or context-specific factors, such as social support or competitive perfectionism (Murphy et al., 1999; Iancheva et al., 2020; Leguizamo et al., 2021).

By understanding the short-term implications of these psychological responses of athletes, their sport context can provide appropriate support and guidance to help them overcome their injuries and prevent future ones (Hausken-Sutter et al., 2021). Additionally, coaches could develop training routines and take advantage of psychological strategies to prevent the use of maladaptive coping strategies. Professionals specialized in sport injury treatment and prevention could benefit from these findings to foster a supportive team environment that emphasizes the importance of social and coaching staff support (Griffin et al., 2021).

As previous studies suggested, the amount and quality of social support needed by injured athletes varies depending on the injury severity and successful completion of milestones in the recovery process. These factors influence the overall experience as more devastating for some athletes than others (Wiese-Bjornstal et al., 1998; Bianco, 2001). In line with the aforementioned research and the narrative of our sample, social support fosters the use of solution-oriented coping strategies. Athletes emphasized the importance of receiving support from health professionals and coaches to continue with their recovery efforts. Conversely, athletes who reported lack of support from healthcare professionals referred to engaging in behavioral risk. Therefore, as it has been remarked in decades of research in the sport psychology field (Martens, 1987; Witt and Dangi, 2018), special attention should be directed to enhancing the relationship between athletes and coaches in general, and injured athletes with healthcare professionals in particular to prevent the first from quitting their sport prematurely (Gómez-Espejo et al., 2022). Aversive emotional responses to injury should be considered a logical part of the injury experience and their presence does not necessarily hinder the recovery process (Guo et al., 2021).

## Limitations

This study applied a cross-sectional design that does not allow to establish causal relationships between the variables studied. The relatively small sample size of this study limits the generalizability of results. Furthermore, the retrospective self-reported data collected might be subject of memory bias or the athletes' unaware intentions to present themselves in a socially desirable way. In line with van Wilgen and Verhagen (2012), further research should consider the perspectives of coaches and healthcare professionals to expand our understanding of the psychological factors involved in the recovery process of athletes. Also, more demographical data such as the participants' experience in their sports discipline, could have been included to expand the comparative analysis of the sample. The authors of this study acknowledge that the analysis procedure could contain subjective elements, and it is possible for other authors to present a different interpretation of the data. These limitations should be taken into consideration when interpreting the results. Future studies using research designs that incorporate both subjective and objective measures are needed to gain a better understanding of the relationship between attributions and coping strategies in the context of sport injuries.

## Conclusions

The coach-athlete relationship creates a supportive environment that facilitates the acquisition and implementation of effective behaviors, resulting in enhanced sport injury recovery outcomes (Mosewich et al., 2013; González-García et al., 2023). Therefore, it is recommended that coaches and health practitioners acknowledge and prioritize the learning of appropriate behavioral rules throughout the recovery process, including educating athletes about myths surrounding medication consumption in long-term injuries. Additionally, coaches should be mindful of the language they use, avoiding imprecise or extreme expressions about competition and sport in general that may predispose athletes to engage in behavioral risk. Athletes' responses to injury and recovery may be negatively affected by language that includes rigid, extreme, or illogical beliefs (Turner, 2016).

These findings highlight the need to standardize interventions for athletes who suffer sport injuries, developing a protocol for prevention, intervention, and support. This protocol should be made available to clubs and federations to minimize the physical and psychological impact of injuries and reduce engagement in behavioral risk not only during their recovery process, but their sport career. Furthermore, athletes could benefit from understanding the role of sport psychology, particularly in the recovery process of sport injuries.

Future research should pay close attention to how coaches, managers, support staff, parents, and athletes use language within training and performance contexts, since it can significantly influence athletes' attributions and coping strategies. Addressing these communication patterns and modifying them might help prevent and manage sport injury processes more effectively (Turner, 2016; Olmedilla-Zafra et al., 2017). Also, future lines of research should consider exploring in depth the concerns expressed about medication, as well as the athletes' interest in receiving psychological support.

The combination of qualitative interviews and the recognition of context-specific situations highlights the significance of understanding athletes' psychological responses beyond a general attributional framework. Taking into account the specific circumstances and context can provide deeper insights to benefit athletes who are at risk of SI. Finally, assessing the attributions and coping strategies of athletes along the different stages of SI could not only benefit athletes themselves, but help reduce memory bias and increase the reliability of coping effectiveness measurements to better address the potential impact of this line of research on applied practice. Further research with larger sample sizes and more rigorous study designs would be needed to confirm these findings and to determine the generalizability of the results to other sport populations and settings.

## Data availability statement

The datasets presented in this study can be found in online repositories. The names of the repository/repositories and accession number(s) can be found at: https://data.mendeley.com/datasets/fwbh2sg395/1.

## Ethics statement

The studies involving humans were approved by the Comitè d'Ètica de la Recerca from Balearic Islands University. The studies were conducted in accordance with the local legislation and institutional requirements. The participants provided their written informed consent to participate in this study.

## Author contributions

FL: Conceptualization, Formal analysis, Methodology, Writing—original draft. AN: Investigation, Methodology, Writing—original draft, Writing —review and editing. EG: Data curation, Formal analysis, Methodology, Writing—original draft. AO: Conceptualization, Methodology, Writing—review and editing. AG-M: Conceptualization, Supervision, Writing—original draft, Writing—review and editing.
